# Dynamic evolution of mitochondrial genomes in Trebouxiophyceae, including the first completely assembled mtDNA from a lichen-symbiont microalga (*Trebouxia* sp. TR9)

**DOI:** 10.1038/s41598-019-44700-7

**Published:** 2019-06-03

**Authors:** Fernando Martínez-Alberola, Eva Barreno, Leonardo M. Casano, Francisco Gasulla, Arántzazu Molins, Eva M. del Campo

**Affiliations:** 1 ICBIBE, Botánica, Facultad de Ciencias Biológicas, Universitat de València, Dr. Moliner 50, 46100-Burjassot, Valencia, Spain; 2 Department of Life Sciences, University of Alcalá, 28805 Alcalá de Henares, Madrid Spain

**Keywords:** Evolution, Plant sciences

## Abstract

Trebouxiophyceae (Chlorophyta) is a species-rich class of green algae with a remarkable morphological and ecological diversity. Currently, there are a few completely sequenced mitochondrial genomes (mtDNA) from diverse Trebouxiophyceae but none from lichen symbionts. Here, we report the mitochondrial genome sequence of *Trebouxia* sp. TR9 as the first complete mtDNA sequence available for a lichen-symbiont microalga. A comparative study of the mitochondrial genome of *Trebouxia* sp. TR9 with other chlorophytes showed important organizational changes, even between closely related taxa. The most remarkable change is the enlargement of the genome in certain Trebouxiophyceae, which is principally due to larger intergenic spacers and seems to be related to a high number of large tandem repeats. Another noticeable change is the presence of a relatively large number of group II introns interrupting a variety of tRNA genes in a single group of Trebouxiophyceae, which includes Trebouxiales and Prasiolales. In addition, a fairly well-resolved phylogeny of Trebouxiophyceae, along with other Chlorophyta lineages, was obtained based on a set of seven well-conserved mitochondrial genes.

## Introduction

The use of organelle genomic information has become a common practice for comparative studies and phylogenetic analyses of entire genomes (phylogenomics). The sequencing of organelle genomes provides valuable information about the evolution of both the organelles and the organisms that carry them. Green photosynthetic eukaryotic organisms include both Chlorophyta and Streptophyta phyla. Chlorophyta comprises unicellular and multicellular green algae, whereas Streptophyta contains both green algae and embryophytes^1^. Initially, morphology and ultrastructural data allowed for distinguishing four classes within Chlorophyta: Chlorophyceae, Prasinophyceae, Trebouxiophyceae and Ulvophyceae, in alphabetical order^2^. Later, molecular data contributed to elucidating the evolution of chlorophytes, corroborating the initial hypothesis of the antiquity of Prasinophyceae, which gave rise to the remaining Chlorophyta classes^3,4^. Regarding this issue, the phylogenetic relationships among chlorophytes remain controversial, especially at higher taxonomic levels (order, class).

Chloroplast genomes are especially attractive for evolutionary studies of photosynthetic eukaryotes. Currently, the number of complete sequences of organellar genomes in the NCBI databases is more than fifteen-fold greater in streptophytes than in chlorophytes (2,492 and 161 are available in the NCBI databases, respectively); among the 161 genomes from chlorophytes, only 56 correspond to mitogenomes. This imbalance is more remarkable among Trebouxiophyceae, since the availability of chloroplast genomes has increased in the few last years (approximately 30 chloroplast genomes are available in the NCBI databases, most of them are published)^1,5,6^. In contrast, almost a dozen mitogenomes are currently available, and some of them were recently published, including those of *Chlorella heliozoae*, *Micractinium conductrix*^7^ and *Botryococcus braunii*^8^.

Trebouxiophyceae have a wide range of lifestyles, including free-living species, endosymbionts of heliozoa (‘Chlorella’-like green algae)^9^, plants (e.g., *Coccomyxa*)^10^, mutualistic or parasitic associations with invertebrates^11^, non-photosynthetic microalgae (e.g., *Helicosporidium* and *Prototheca*)^12^ and symbionts of fungi (e.g., *Trebouxia, Asterochloris, Symbiochloris, Myrmecia* and others)^13–15^.

Within the Trebouxiophyceae, 22 genera are known to be involved in symbiosis with lichen thalli^15^. However, there is no completely sequenced mitochondrial genome from any lichen microalga, since only four regions of the mtDNA of *Trebouxia aggregata* are available in GenBank (accessions EU123944, EU123947, EU123948 and EU123949). The genus *Trebouxia* is one of the most species-rich microalgal genera, comprising non-motile coccoid green algae that are present in approximately one half of all lichens^15^. The initial number of *Trebouxia* species formally described on the basis of phenotypic characters was approximately 30^16^. This number has increased in recent years after the application of phylogenetic species concepts (e.g.^17–19^). However, the lack of closed complete genomes that can be used as references has hindered the systematic study of the molecular evolution of the members of the *Trebouxia* genus. *Trebouxia* sp. TR9 is a phycobiont of the lichen *Ramalina farinacea* (L.) Ach., which has been extensively studied in recent years in relation to many ecological and physiological traits^20–26^. However, molecular analyses of this *Trebouxia* species have been restricted to a few molecular markers from both nuclear and chloroplast genomes (e.g.^26–30^) without consideration of the mitochondrial genome. In this study, we report the complete sequence of the mitochondrial genome of *Trebouxia* sp. TR9, determined from high-throughput Roche 454 pyrosequencing. We compare its structure, organization and gene content with other mitochondrial genomes reported for Trebouxiophyceae and other Chlorophyta microalgae and provide a phylogenetic reconstruction on the basis of seven selected mitochondrial genes (*cob*, *cox1*, *nad1*, *nad2*, *nad4*, *nad5* and *nad6*).

## Results

### Structural features of the completely assembled mtDNA of *Trebouxia* sp. TR9

**Figure 1:**
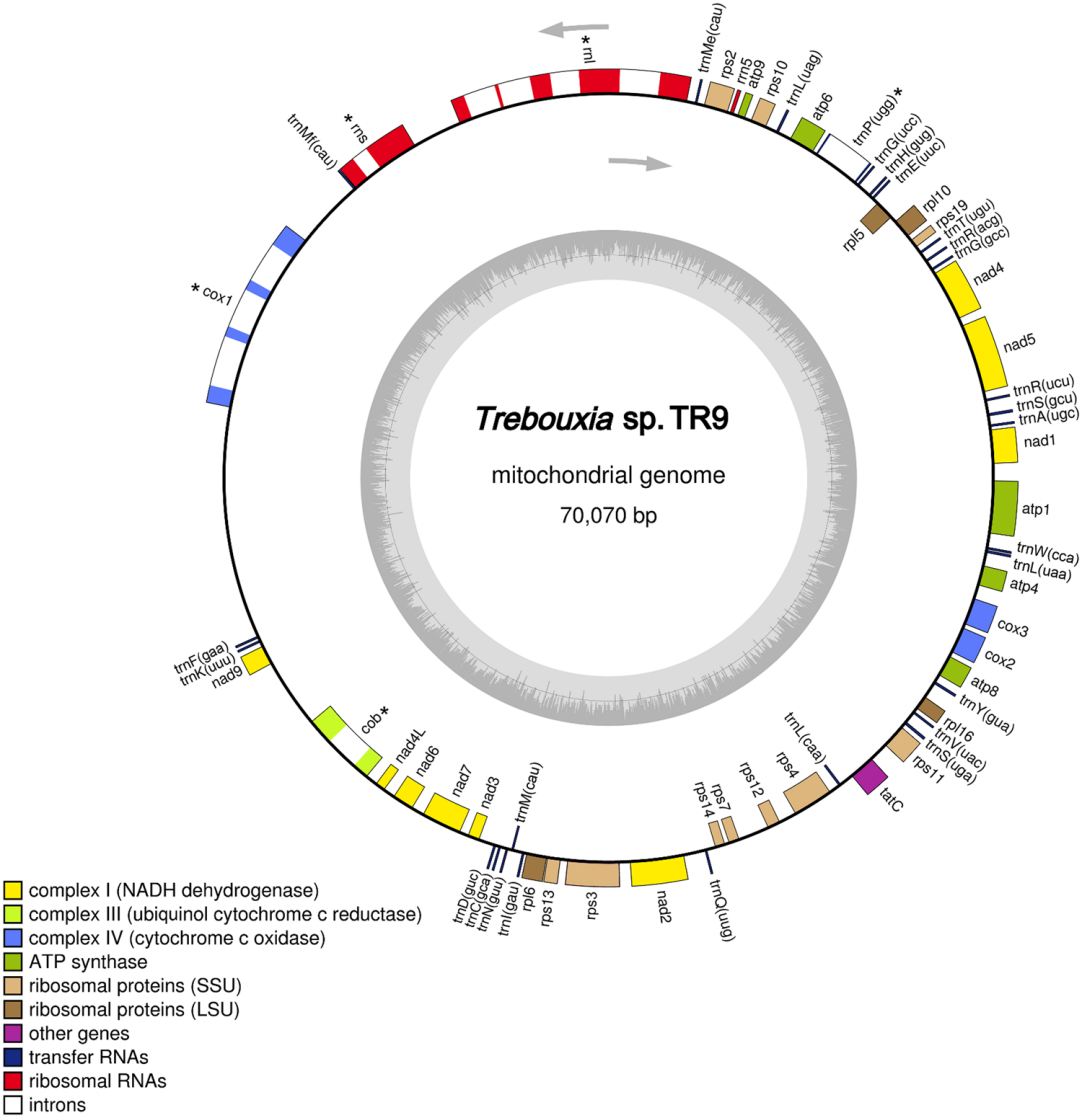
Gene map of the complete mitochondrial genome of the microalga *Trebouxia* sp. TR9. Genes shown inside the circle are transcribed clockwise, and genes outside are transcribed counter clockwise. Asterisks indicate genes with introns.

The mitochondrial genome (mtDNA) of *Trebouxia* sp. TR9 (Fig. 1) is a circular molecule of 70,070 bp with a GC content of 32.7% and a total of 67 genes. Thirty-three genes encoded conserved proteins, including nine subunits of the electron transport complex I (*nad1*, *nad2*, *nad3*, *nad4*, *nad4L*, *nad5*, *nad6*, *nad7* and *nad9*), one subunit of complex III (*cob*), three subunits of complex IV (*cox1*, *cox2* and *cox3*), five F0 subunits of the ATP-synthase complex (*atp1*, *atp4*, *atp6*, *atp8*, and *atp9*), fourteen ribosomal proteins: ten for the small ribosomal subunit (*rps2*, *rps3*, *rps4*, *rps7*, *rps10*, *rps11*, *rps12*, *rps13*, *rps14* and *rps19*) and four for the large ribosomal subunit (*rpl5*, *rpl6*, *rpl10* and *rpl16*), the *TatC* membrane protein, and four genes encoding putative LAGLIDADG homing endonucleases (LHEs). In addition, 27 tRNA genes and three genes for ribosomal RNAs (*rrnl, rrns* and *rrn5*) were identified in the *Trebouxia* sp. TR9 mtDNA. Regarding the tRNAs, a total of 26 tRNA genes were identified with RNAweasel and tRNAscan-SE, whereas with ARAGORN, we found 27 tRNAs, including an additional *trnP* (ugg). Three tRNA genes with different sequences and the same anticodon (cau) were identified for tRNA-Met. Three tRNA genes with different sequences and anticodons were found for tRNA-Leu, and two tRNA genes with different sequences and anticodons were found for tRNA-Gly, tRNA-Ile, tRNA-Arg and tRNA-Ser. For the remaining 13 tRNAs, only one gene each was found. The additional tRNA gene found with ARAGORN corresponded to tRNA-Pro (ugg) spanning from positions 9,751 to 11,174, with a group II intron of 1,350 bp predicted with RNAWEASEL. Regarding intron content, a total of ten introns were identified within the mtDNA of *Trebouxia* sp. TR9 with RNAweasel. Nine of them were group I introns, and only one belonged to group II (Fig. 1). Group I introns were located within the genes *rrnL*, *rrnS*, *cob* and *cox1*. Most of them belonged to group IB (within the genes *cox1* and *rrnL*), followed by group IA (within genes *rrnS* and *rrnL*) and a single intron of group ID (within the gene *cob*). Intron sizes ranged from 500 to 1,443 bp within the genes *rrnS* and *cox1* (third intron), respectively. Only introns within the genes *cob* and *cox1* included open reading frames (ORFs) encoding homing endonucleases (HEs), with either a single or two LAGLIDADG motifs in each of them. As stated above, a group II intron was found in the gene coding tRNA-Pro (ugg).

### Phylogenetic analyses of Trebouxiophyceae and other Chlorophyta lineages based on seven mitochondrial genes

**Figure 2:**
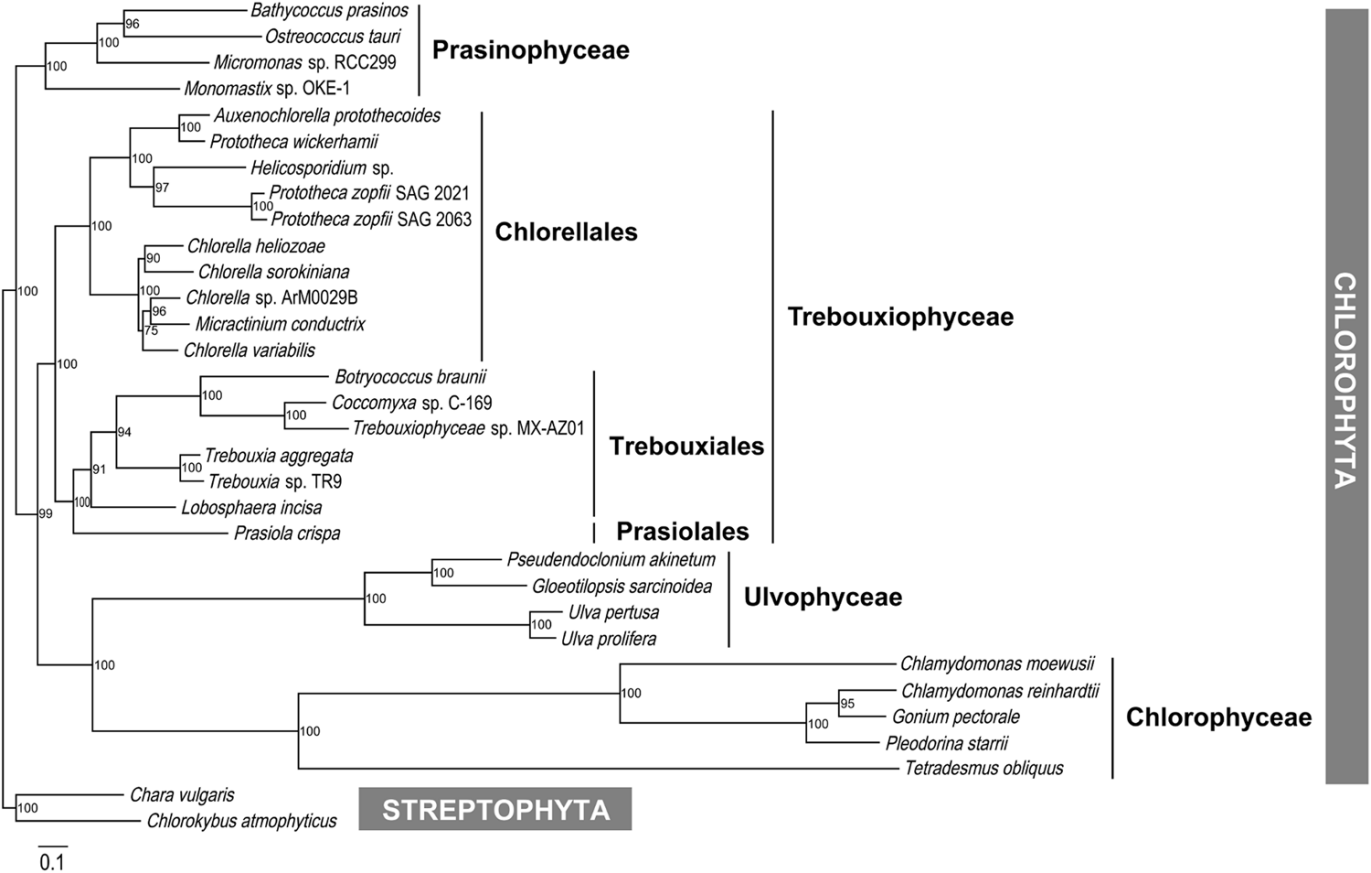
Phylogram based on the sequence analysis of seven mitochondrial genes from 32 green algal species (Table S1). Bootstrap values are indicated in the nodes.

Here, we present a phylogenetic reconstruction of chlorophytes (Fig. 2), including species from different divisions and two streptophytes as outgroups, *Chlorokybus atmophyticus* and *Chara vulgaris* (see Table S1 for accessions). All analyses were based on a nucleotide sequence alignment of 9,032 bp, including the sequences without introns of seven mitochondrial genes (*cob, cox1, nad1, nad2, nad4, nad5* and *nad6*), which are conserved among all the studied chlorophytes. The phylogram in Fig. 2 shows four major clades: the first clade included the Prasinophyceae, the second clade included the Trebouxiophyceae, the third clade included the Ulvophyceae, and the fourth clade included the Chlorophyceae. Our phylogenetic reconstruction shows that *Trebouxia* sp. TR9 is closely related to *Trebouxia aggregata*, another lichen phycobiont. The two lichen microalgae were included within a sub-clade, along with *Botryococcus braunii*, *Coccomyxa* spp., *Lobosphaera incisa*, *Micractinium conductrix* and *Prasiola crispa*. This sub-clade II, including Trebouxiales and Prasiolales, is a sister of another sub-clade I that includes Chlorellales.

### Gene content of the mtDNAs from Trebouxiophyceae and other chlorophytes

**Figure 3:**
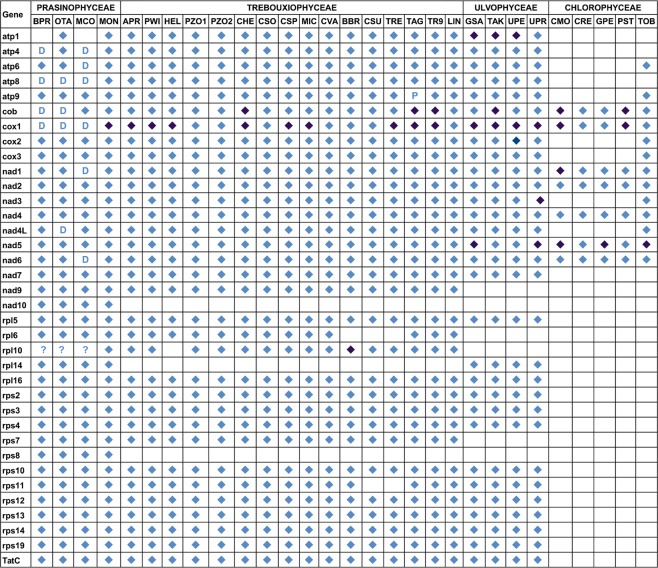
Gene repertoires of the mtDNAs from the green algal mtDNAs examined in this study. Diamonds indicate the presence of a standard gene. A “D” denotes gene duplications. Light and dark blue diamonds indicate the presence or absence of introns, respectively. “P” and “?” indicate a partial sequence and uncertainty, respectively.

**Figure 4:**
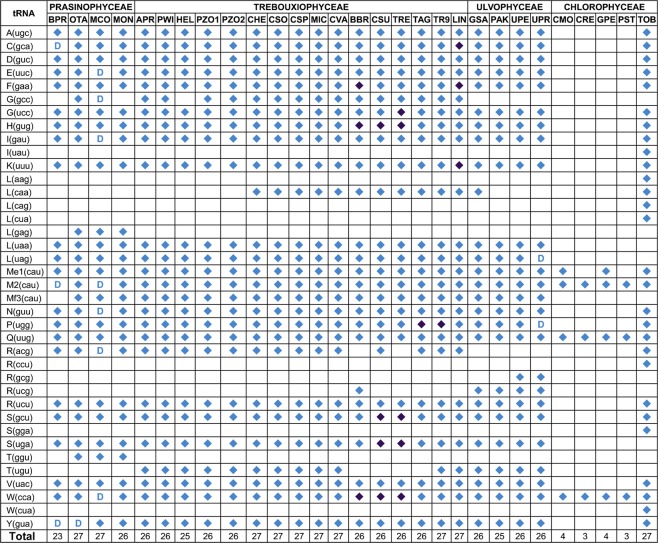
Transfer RNA repertoires of the mtDNAs from the green algal mtDNAs examined in this study. A diamond or a “D” indicates the presence of a standard gene if it was duplicated. The presence or absence of introns is indicated by light and dark blue diamonds, respectively.

Figures 3 and 4 show the repertoire of genes coding conserved proteins and tRNAs, respectively, in a number of Chlorophyta algae belonging to different classes (Prasinophyceae, Trebouxiophyceae, Ulvophyceae and Chlorophyceae). At least 26 genes coding for proteins were shared by the studied species belonging to Prasinophyceae, Trebouxiophyceae and Ulvophyceae. Conversely, the studied Chlorophyceae, except *Tetradesmus obliquus*, showed extensive gene loss (most of them coding for ribosomal proteins and tRNAs). Several genes seemed to be lost in certain species within an algal class. For instance, the *rpl6* and *rps11* genes are present in all the studied Trebouxiophyceae except *Coccomyxa* sp. C169 and *Trebouxiophyceae* sp. MX-AZ01. Other genes seemed to be retained in specific algal classes, as is the case of the *rpl10* gene.

In this study, we identified the *rpl10* gene in the mitochondrial genome of all the studied Trebouxiophyceae, Prasinophyceae, *Micromonas* sp., *Monomastix* sp. and *Ostreococcus tauri* (Table S2). The hypothetical mitochondrial ribosomal L10 proteins would have variable sizes ranging from 510 to 867 aa. Additionally, the *rpl10* gene was located downstream of *rps19* in all the studied Trebouxiophyceae except for *Auxenochlorella protothecoides* and *Prototheca wickerhamii*, in which *rpl10* mapped downstream of *rps10*. The genes downstream of *rpl10* were more variable, including genes encoding proteins, rRNAs and tRNAs. *Botryococcus braunii* had a group II intron of 2,598 bp in the *rpl10* gene (positions 1,288 to 3,885 in the nucleotide sequence with accession number NC_027722), which was predicted with the program RNAweasel. This intron was the only group II intron within a protein-coding gene found in the Trebouxiophycean algae analysed in this study.

### Comparative analysis of the structure of the mtDNAs from Trebouxiophyceae and other chlorophytes

**Figure 5:**
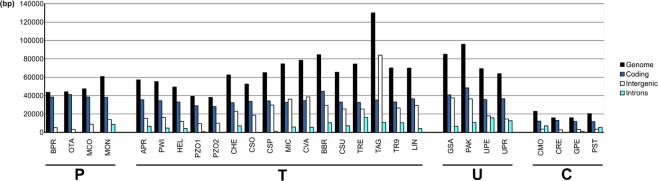
Total lengths (bp) of coding, intronic, and intergenic sequences in the chlorophyte mtDNAs examined in this study. Species names are abbreviated as in Table S1. The systematic classification is indicated at the bottom (P: Prasinophyceae, T: Trebouxiophyceae, U: Ulvophyceae, C: Chlorophyceae). Data for *Trebouxia aggregata* were obtained from partial sequences (accessions EU123944, EU123947, EU123948 and EU123949).

A comparison of the structure of the mtDNAs from different chlorophytes (Fig. 5) showed strikingly variable sizes among Trebouxiophyceae, ranging from 38,164 bp in *Prototheca zopfii* SAG 2063 to more than 130,000 bp that results from the sum of the partial sequences of *Trebouxia aggregata* available in GenBank. The complete mitochondrial genome of *Trebouxia* sp. TR9 had identical repertoires of genes coding for proteins and tRNAs as the lichen-symbiont alga *Trebouxia aggregata*, whose mitogenome has not been completely sequenced (except *trnT* (ugu) and a partial sequence of *atp9*, probably due to the incompleteness of the available sequences) (Figs 3 and 4). Such repertoires were approximately the same as that of free-living Trebouxiophyceae. Thus, the symbiotic association with the mycobiont does not seem to have any impact on the gene content of the mtDNA in the two studied *Trebouxia* species.

The remarkable enlargement of the mtDNAs in Trebouxiophyceae and Ulvophyceae with respect to other chlorophytes was due to the presence of more introns and larger intergenic spacers (Fig. 5). The most extreme difference in the mitogenome size (of at least 59,986 bp) was found between the two closely related *Trebouxia* microalgae. This difference is mostly due to non-coding regions, which included 37,029 bp and 94,900 bp in *Trebouxia* sp. TR9 and *T. aggregata*, respectively, while the coding regions were quite similar: 33,041 bp and 35,156 bp in *Trebouxia* sp. TR9 and *T. aggregata*, respectively. A more detailed picture of these differences can be observed in Fig. S1, which shows the genetic maps of four regions of the mtDNA of *T. aggregata* (accessions EU123944, EU123947, EU123948 and EU123949) and their counterparts in *Trebouxia* sp. TR9. This figure depicts important differences in the total lengths due to longer intergenic regions in *T. aggregata*, which contrasts with the more compact structure observed in *Trebouxia* sp. TR9. Furthermore, this difference was not due to any large insertion/deletion at a specific part of the genome but to small increases or decreases in every intergenic region. Figure S1 also shows high synteny between the mtDNAs from these two *Trebouxia* species. The whole-genome alignment of the *Trebouxia* sp. TR9 mtDNA along with other chlorophytes (Fig. S2) showed high conservation of many coding regions, along with remarkable rearrangements, using the steptophyte *Mesostigma viride* as a reference (accession NC_008240). The most compact and smallest mitochondrial genomes belonged to the Chlorophyceae, which showed intensive gene loss (Fig. 5).

**Figure 6:**
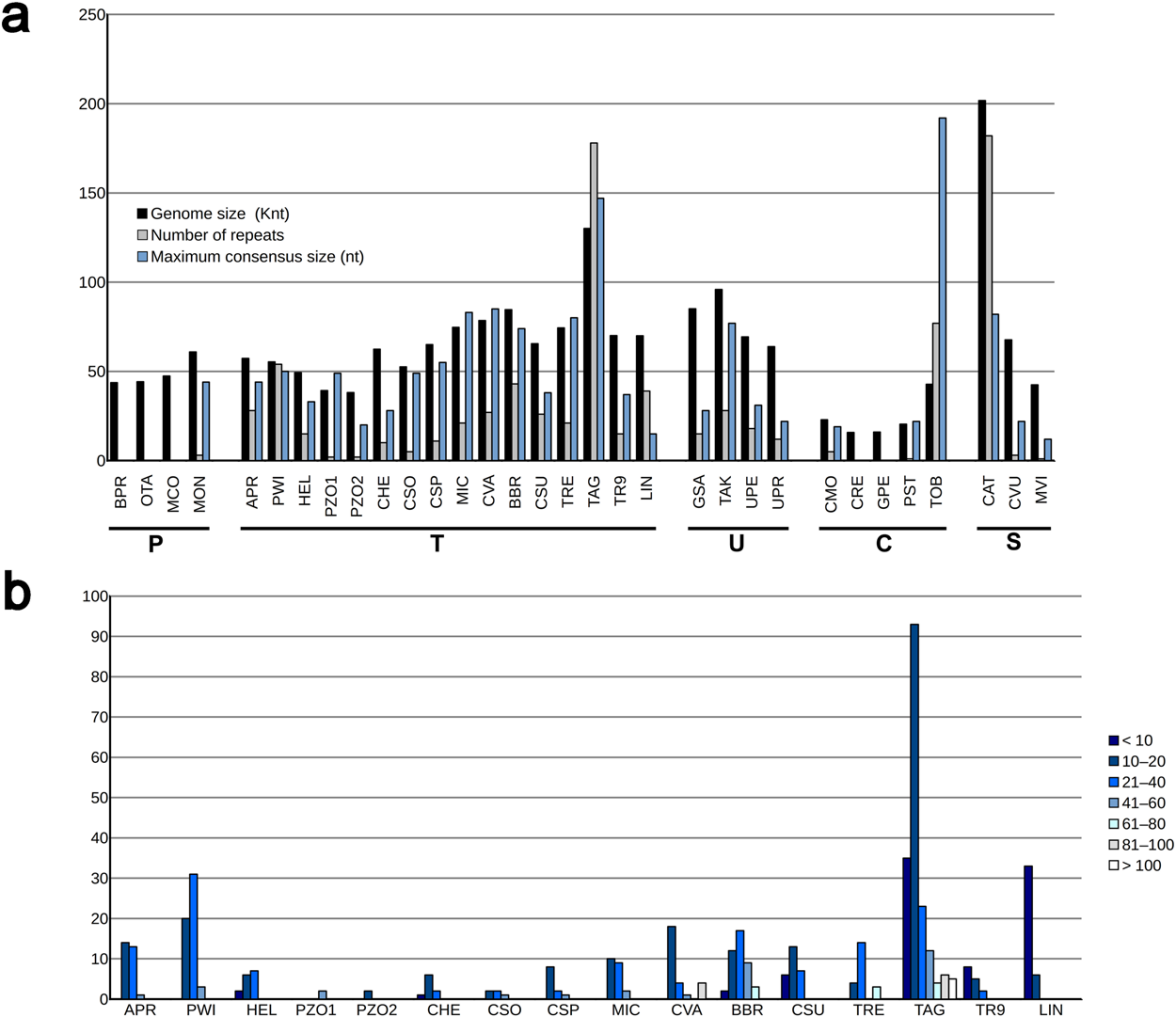
Tandem repeats in the green algal mtDNAs examined in this study. (**a**) Genome sizes, number of repeats found and maximum consensus size. (**b**) Frequency of tandem repeats by length in Trebouxiophyceae. The systematic classification is indicated at the bottom (P: Prasinophyceae, T: Trebouxiophyceae, U: Ulvophyceae, C: Chlorophyceae, S: Streptophyta).

To determine if the expanding/contracting regions were related to the presence of repeats, we searched for tandem repeats (*trs*) using the program “Tandem repeats finder”. Tandem repeats are mainly characterized by the number of copies and their consensus size (the length of the repeat unit). Our results (Fig. 6a) indicated that *Trebouxia* sp. TR9 had one of the lowest numbers of *trs* of the studied Trebouxiophyceae, along with *Chlorella heliozoae*, *Chlorella sorokiniana*, *Chlorella* sp., *Helicosporidium sp*. and *Prototheca zopfii*, all them with less than 15 *trs*. The remaining studied Trebouxiophyceae had a number of *trs* between 20 and 60, except for *Trebouxia aggregata*, which displayed the largest number of *trs* of 178. These *trs* were mostly located within intergenic spacers (169 out of 178). Figure 6b shows the distribution of the consensus size for all *trs* found in the mtDNAs from Trebouxiophyceae. The consensus size of the *trs* was highly heterogeneous among the studied Trebouxiophyceae (Fig. 6b). Generally, the *trs* with larger consensus sizes were found in mtDNAs with a high number of *trs*. In our analysis, we found large *trs* of more than 140 bp of period size in only two chlorophytes belonging to different classes: *Trebouxia aggregata* and *Tetradesmus obliquus*, with maximum consensus sizes of 147 and 192, respectively (Fig. 6a). Notably, within each algal class, the species with the largest mtDNA also has the highest number and consensus size of *trs* (e.g., *Monomastix* sp. within the Prasinophyceae, *Trebouxia aggregata* among the Trebouxiophyceae, *Tetradesmus obliquus* among the Chlorophyceae and *Chlorokybus atmophyticus* among the Streptophyta). The loosely packed mitochondrial genome of *T. aggregata* and the more compact mitochondrial genome of *Trebouxia* sp. TR9 may be a consequence of either an expansion or contraction of intergenic regions, respectively. Such expansion/contraction of intergenic regions may have occurred by gain/loss of tandem repeat units, i.e., by varying the number of copies rather than by insertion/deletion of fragments of different sizes.

### Diversity of intron content of the Chlorophyta algae mitochondrial genomes

**Figure 7:**
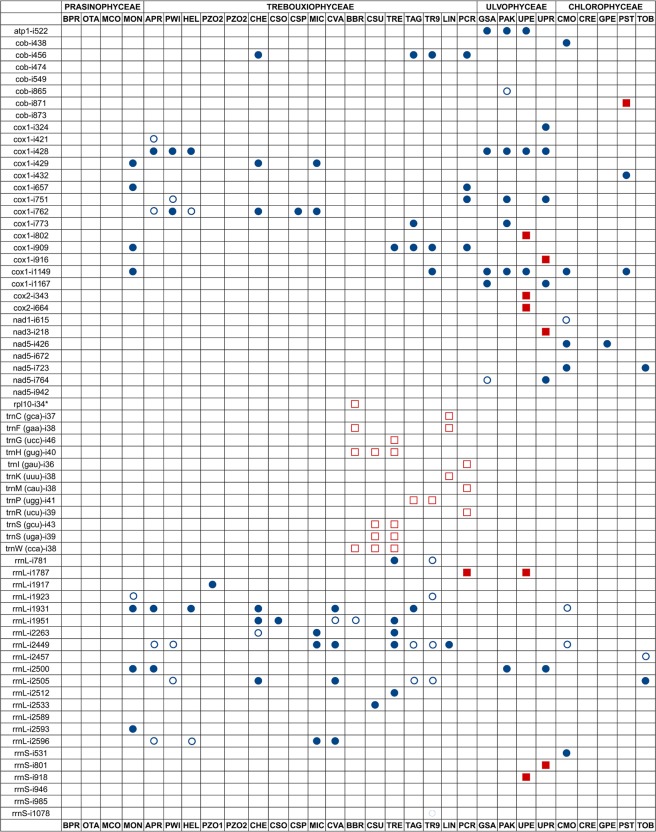
Distribution of introns among the green algal mtDNAs examined in this study. Blue circles and red squares indicate the presence of either a group I or group II intron, respectively. Filled symbols and empty symbols denote introns containing or lacking ORFs, respectively. The insertion positions are provided after the gene names and are preceded by an “i”. Positions are relative to *Mesostigma viride* mtDNA (for protein-coding and tRNA genes) and *Escherichia coli* (for rRNA genes).

The coding regions of many genes are interrupted by introns in a variety of genetic systems and organisms. To date, four main types of introns have been distinguished based on their splicing mechanism: spliceosome introns, nuclear and archaeal tRNA introns, group I introns and group II introns. As far as we know, our study provides the first compilation of data about the variety of introns, including both group I and group II introns, in the mitochondrial genomes from a number of chlorophytes (Fig. 7). A total of 91 introns were found in the mtDNA of the studied Trebouxiophyceae: 66 group I and 25 group II introns. Most group I introns were found either in certain protein-coding genes (*cob* and *cox1*) or the gene encoding the LSU rDNA. Group II introns were mostly found within genes coding certain tRNAs, except two introns inserted within the *rpl10* and *rrnL* genes of the microalgae *B. braunii* (Trebouxiales) and *P. crispa* (Prasiolales), respectively. In other chlorophytes analysed in this study belonging to other classes different from Trebouxiophyceae, a total of 77 introns was observed: 42 group I and 35 group II introns. A number of ORFs were coded within introns and corresponded to putative LAGLIDADG homing endonucleases in the case of group I introns and maturases or reverse transcriptases in the case of group II introns.

As stated above, we identified the *rpl10* gene of *B. braunii* as the first protein-coding gene bearing a group II intron in the mitochondrial genome of a Trebouxiophyceae (Table S2). The gene for the ribosomal protein L10 (*rpl10*) is present in a wide diversity of land plants and mitochondrial genomes of algal streptophytes. However, this gene has remained unidentified and largely unannotated in the records of sequenced mitochondrial genomes from green algae. In most cases, this gene actually corresponded to conserved ORFs of unknown function. In other cases, this gene has remained unannotated (Table S2).

One of the most striking features of the mtDNAs analysed in our study was the relatively high number of group II introns disrupting a variety of genes coding tRNAs in a specific group of Trebouxiophyceae (Fig. 7). Indeed, a total of 12 genes coding tRNAs had a group II intron: *trnC* (gca), *trnF* (gaa), *trnG* (ucc), *trnH* (gug), *trnI* (gau), *trnK* (uuu), *trnM* (cat), *trnP* (ugg), *trnR* (ucu), *trnS* (gcu), *trnS* (uga) and *trnW* (cca). Some of these introns were unannotated in their respective genomic sequences (Table S2). All the Trebouxiophyceae with introns in genes coding tRNAs belonged to clade II (Fig. 2), including Trebouxiales and Prasiolales. tRNAs are fundamental components of the translation machinery and in the regulation of gene expression and several other biological processes.

Only two *Trebouxia* algae analysed in this study had a group II intron within the gene coding tRNA-Pro (ugg). In *T. aggregata*, this intron has not been annotated (position 6,937 to 8,915 in the sequence, GenBank accession number EU123949). Another *Trebouxia* phycobiont associated with the lichen *Rhizocarpon geographicum* (*Trebouxia* sp. RG in this study) showed the partial sequence of an intron within the *trnP* (ugg) gene (position 1 to 156 in the sequence, accession number JN847694). In the three *Trebouxia* algae, the *trnP* gene with a group II intron had a conserved position upstream of the *atp6* gene. Comparison of the intergenic spacer between the *trnP* and *atp6* genes in the three *Trebouxia* algae showed different lengths, including 115, 326 and 1,152 bp in *Trebouxia* sp. TR9, *Trebouxia* sp. RG and *T. aggregata*, respectively. Interestingly, a total of six *trs* were found in this spacer in *T. aggregata*, one of them with a period size of 95, whereas no *trs* were found in the other two *Trebouxia* algae with shorter intergenic spacers. This finding reinforces the notion of an enlargement of the mitochondrial genome of *T. aggregata* due to the duplication of sequences within intergenic spacers rendering the observed high number of *trs*. The *trnP* (ugg) gene without any introns was also present in all the studied chlorophytes except for those belonging to Chlamydomonadales, which lack this gene (Fig. 4).

## Discussion

The phylum Chlorophyta comprises morphologically and ecologically diverse green algae, which have traditionally been included within three major clades: Ulvophyceae, Trebouxiophyceae, and Chlorophyceae (UTC clade). Moreover, the replacement of “UTC clade” with the term “core Chlorophyta” has been proposed to indicate the previous UTC taxa plus additional classes^1,4,31^. The class Chlorophyceae is considered to be a monophyletic group^32–34^. However, the monophyly of Ulvophyceae and Trebouxiophyceae is not strongly supported by several molecular studies, and their polyphyly has been suggested in several publications^29,31,34^. The monophyly/polyphyly of Trebouxiophyceae depends on the selected sequences and taxon sampling. In some studies, Chlorellales is placed in a clade independent of other Trebouxiophyceae (e.g., Trebouxiales)^5,6,31,34,35^, whereas other studies support the monophyly of Trebouxiophyceae^36,37^. The topology of the phylogram obtained here (Fig. 2) is consistent with the placement of Chlorellales with Trebouxiales in a single clade, as proposed by other phylogenetic reconstructions based on a higher number of mitochondrial genes^7^ and plastid genes^7,35,36,38^; however, it is noteworthy that this reconstruction is based on a rather limited variety of algal groups. Some authors state that sampling across different algal groups is a prerequisite for deriving a reliable phylogenetic classification of the core Chlorophyta^31^. Moreover, in several phylogenetic studies based on chloroplast sequences, the inferred topologies were dependent upon the data set and the method of analysis, differing mainly with respect to the relative positions of the major lineages in the core Chlorophyta^39^.

As previously stated, the symbiotic association does not seem to influence the gene content of the mtDNA in the two studied *Trebouxia* species. This observation contrasts with the reduction in genome content observed in other symbiotic relationships, such as bacterial endosymbionts of insects^40^. However, our findings are consistent with the absence of organellar genomic reduction observed in some green algae involved in other types of symbiotic relationships, which tend to have larger mtDNAs^7^, and suggest that symbiosis may promote larger mtDNAs. In addition, the gene content of the mtDNA from Trebouxiophyceae was similar to that of several streptophytes^38^, indicating their conservation during the evolution from a common ancestor.

The hypothetical expansion or contraction of intergenic regions in the mitogenomes of the studied *Trebouxia* algae by gain/loss of tandem repeat units may have occurred in other algal groups, such as Streptophyta algae. Within this algal group, *Chlorokybus atmophyticus* has a large mtDNA (201,763 nt) with very extended intergenic spacers^41^ and 182 *trs*, whereas *Mesostigma viride* has a more compact mtDNA (42,424 bp)^42^ and a single predicted *tr*. In this line, we observed a certain parallelism with previous studies of obligate intracellular livestock pathogens^43^. In the referred studies, the bacterium *Ehrlichia ruminantium* displayed a lower coding ratio due to unusually long intergenic regions related to an active process of genome expansion/contraction. This process was targeted at trs in non-coding regions, based on the addition or removal of 150-bp tandem units and seemed to be specific to *E. ruminantium*. This finding agrees with previously proposed mechanisms of tr deletion or amplification through DNA slippage^44^. Moreover, *E. ruminantium* seemed to be capable of rapidly undergoing genomic rearrangements upon exposure to novel environmental conditions^43^. It has been proposed that mitochondrial genomic architecture is shaped by two types of mtDNA repair^45^: (i) within genes, in which gene conversion would maintain low mutation rates, and (ii) within non-coding regions, in which expansion(s) and rearrangements may be explained by break-induced replication (BIR). Both processes can explain the low mutation rates in coding sequences and the striking expansions of non-coding sequences. The same argument has been proposed for the mitochondrial genome from several *Dunaliella* species^46^, which have undergone massive levels of mitochondrial genomic expansion. Moreover, BIR within organelle systems is known to be inaccurate and cause rearrangements and expansions in *Arabidopsis thaliana*^47^. It is plausible that the intergenic regions in the *Trebouxia* mitochondrial genomes would also be shaped via BIR. This mechanism may explain the expansion and disarray observed in the intergenic regions of the mitogenomes from *T. aggregata* in relation to those from *Trebouxia* sp. TR9.

Several studies indicated that plants are the only group of eukaryotes other than *Reclinomonas* (Excavata) that still retain the gene *rpl10* in their mitochondrial genomes^38,48,49^. In this study, we found retention of the *rpl10* gene in the mitochondrial genome of representatives of certain Chlorophyta classes (e.g., Prasinophyceae and Trebouxiophyceae) and its absence in other classes (e.g., Chlorophyceae and Ulvophyceae). These observations, along with its possible pseudogenization in some lineages, are consistent with the model of evolution of the *rpl10* gene in plants proposed by Kubo and Arimura^49^. According to this model, this gene was originally in the mitochondrial genomes. Then, it was lost from most eukaryotic lineages except plants. However, certain plant lineages lack *rpl10* in their mitochondrial genomes and have a nuclear-encoded *rpl10* because of the duplication of the *rpl10* gene transferred from the chloroplast to the nucleus. This copy of *rpl10* seems to functionally compensate for the lack of the mitochondrial *rpl10* gene in any subcellular compartment. This model remains to be demonstrated in chlorophytes.

As far as we know, our study provides the first compilation of data about the variety of introns, which includes group II introns disrupting tRNA genes, in a specific algal group within Trebouxiophyceae (Fig. 7). Introns disrupting tRNA genes were found in a variety of forms and different genetic systems in all the three kingdoms of life, being particularly abundant in archaeal and eukaryotic genomes^50^. Currently, there is a certain controversy on the origin of tRNA introns. An “intron-first” hypothesis suggests that a large part of introns present in all primordial tRNA genes have been lost during evolution. After intron loss, the two halves were joined in the genome, rendering an intron-less tRNA gene^51^. Alternatively, the “intron-late” hypothesis suggests the insertion of introns after the establishment of primordial tRNA genes^52^. The existence of split tRNAs is consistent with the second hypothesis^51^. In our study, several pieces of evidence for the gain of a modern intron by tRNA genes can be observed. First, the presence of introns within tRNA genes is restricted to a single clade among Trebouxiophyceae (clade II in Fig. 2). Moreover, in the case of the *trnP* (ugg) gene, which was exclusively found in *Trebouxia* microalgae, mature parts of tRNA-Pro (ugg) are highly homologous to those of the same tRNA from other related Trebouxiophyceae. Thus, the *trnP* (ugg) gene from the common ancestor of *Trebouxia* algae likely acquired its introns over the course of evolution. A parallel picture can be observed in other intron-bearing tRNA genes. For instance, the intron of the *trnH* (gug) and *trnW* (cca) genes was probably acquired by the common ancestor of these three Trebouxiophyceae: *B. braunii*, *Coccomyxa* sp. C-169 and *Trebouxiophyceae* sp. MX-AZ01. Similarly, the intron of the *trnS* (gcu) and *trnS* (uga) genes was probably acquired by the common ancestor of *Coccomyxa* sp. C-169 and *Trebouxiophyceae* sp. MX-AZ01. In this scenario, the relatively recent intron gain by tRNA genes, which was found in a single algal species in this study, might be the result of a modern acquisition rather than a loss during evolution [e.g., *trnC* (gca) and* trnK* (uuu) genes in *L. incisa*; trnG (ucc) gene in *Trebouxiophyceae* sp. MX-AZ01; *trnI* (gau), *trnM* (cau) and *trnR* (ucu) genes in *B. braunii*].

The structural analyses of the mitochondrial genome of Trebouxiophyceae and other Chlorophyta algae reported in this study contribute considerably to understanding the evolution of the mitochondrial genomes in the most ancestral photosynthetic eukaryotes. Our investigation stresses the importance of providing new sequences of mitochondrial genomes of green algae to find new features that may be crucial to establishing evolutionary patterns in different algal lineages and to more precisely delineate such lineages based on phylogenetic analyses.

## Methods

### Phycobiont isolation and culture conditions

*Trebouxia* sp. TR9 was isolated from the lichen *Ramalina farinacea* (L.) Ach.^53^ and cultured in Bold 3N medium^54^ in a growth chamber at 15 °C under a 14-h/10-h light/dark cycle (lighting conditions: 25 μmol m^−2^ s^−1^).

### DNA isolation and sequencing and genome assembly and annotation

DNA extraction and purification were performed according to the protocol used by Ausubel *et al*.^55^. The purified DNA was sequenced using 454 GS FLX Titanium technology (454 Life Sciences, Roche, Basel, Switzerland) at Lifesequencing facilities (Parc Cièntific, Universitat de València, Spain). The 454 pyrosequencing reads were assembled using Mira assembly software^56^. Contigs corresponding to the mitochondrial genomes were selected using BLASTn, BLASTx and tBLASTx^57^ against a local database of mitochondrial genomes from Viridiplantae built from the NCBI nucleotide databases. To connect the different contigs and corroborate the genome circularity, a number of primers were designed (Table 1). PCR was performed in a 96-well LabCycler (SensoQuest Biomedizicnische Elektronik) using EmeraldAmp GT PCR Master Mix (Takara Bio Inc., Shiga, Japan). PCR products were purified using Illustra GFX PCR DNA (GE Healthcare Life Science, Buckinghamshire, England) and sequenced with an ABI 3100 Genetic Analyzer using an ABI BigDyeTM Terminator Cycle Sequencing Ready Reaction Kit (Applied Biosystems, Foster City, California). Most of the genes and open reading frames (ORFs) were identified using the MFannot organelle genome annotator (http://megasun.bch.umontreal.ca/cgi-bin/mfannot/mfannotInterface.pl). The unannotated *rpl10* genes were identified using BLAST tools. Motifs for both RPL10 proteins and homing endonucleases were found with BLAST and MotifSearch available at https://www.genome.jp/tools/motif/. tRNA genes were localized using RNAweasel^58^, tRNAscan-SE^59^ and ARAGORN^60^.

**Table 1:** List of primers used for DNA amplification and sequencing.

Name	Sequence	Position	Length	Direction
MT_61K_769_F	AGTTTACGGAATTATAACAGCG	776–797	22	forward
MT_61K_1838_R	TACGTTGATTTAGCAAACCAATG	1823–1845	23	reverse
MT_61K_23618_F	AGTAGAGACACAACATCATTAAC	23192–23214	23	forward
MT_61K_24958_R	GAGCTGACGACAGCCATG	24521–24538	18	reverse
MT_61K_60869_R	GAAAGTGGCTCTTCCAGCA	58242–58260	19	reverse
MT_61K_59310_F	TGTGTTTACCTATTTCACCAAG	59759–59780	22	forward
MT_10K_431_F	ACACCTAGTTGGTATTGCTTTG	60470–60491	22	forward
MT_10K_654_R	GGTGTTTGAAAGATAGACTGCA	60690–60711	22	reverse
MT_10K_9453_F	GCATATCGTCAAATGTCATTG	69290–69310	21	forward
MT_10K_9858_R	CAAGTATTGAGTAGCGGCGT	69693–69712	20	reverse

### Phylogenetic analyses

Phylogenetic reconstructions were performed based on seven mitochondrial genes from 32 algal species (see Table S1 for accessions). Alignments were performed with Muscle^61^ with Geneious R10^62^ and trimmed with GBLOCKs^63^, with options for less stringent selection. The less stringent selection of blocks allowed for smaller final blocks, gap positions within the final blocks and less strict flanking positions.

For the maximum-likelihood (ML) analyses, the concatenated nucleotide matrix of 32 taxa and 9,032 bp were analysed with the GTR + G + I model of nucleotide substitution, which was selected according to the automatic model selection of PhyML^64^. Data sets were subjected to ML with PhyML^64^. Bootstrap probabilities^65^ were calculated to estimate the robustness of the clades from 100 replicates in the data. The consensus tree was drawn with FigTree^66^.

### Additional analyses

Whole-genome alignments were performed with MultiPipMaker^67^. Gene maps were constructed with Geneious R10^62^. Tandem repeats were found using the program Tandem repeats finder^68^.

### Accession codes

The complete mitochondrial genome sequence generated in this study has been deposited under the GenBank accession number MH917293.

## Supplementary information

Supplementary Information
