# Supplementary material for: Dynamic evolution of mitochondrial genomes in Trebouxiophyceae, including the first completely assembled mtDNA from a lichen-symbiont microalga (Trebouxia sp. TR9)
Source: Sci Rep. 2019 Jun 3;9:8209. doi: 10.1038/s41598-019-44700-7 (PMC6547736; doi:10.1038/s41598-019-44700-7)
Supplement: Supplementary file 1 — Supplementary Information [file 41598_2019_44700_MOESM1_ESM.pdf]

Supplementary Information: Dynamic evolution of mitochondrial genomes in Trebouxiophyceae, including the first completely assembled mtDNA from a lichen-symbiont microalga (*Trebouxia* sp. TR9)

Fernando Martínez-Alberola[1], Eva Barreno[1], Leonardo M. Casano[2], Francisco Gasulla[2], Arántzazu Molins[1] and Eva M. del Campo[2]

[1]ICBIBE, Botánica, Facultad de Ciencias Biológicas, Universitat de València, Dr. Moliner 50, 46100-Burjassot, Valencia, Spain

[2]Department of Life Sciences, University of Alcalá, 28805, Alcalá de Henares (Madrid), Spain

*Corresponding author: Eva María del Campo. Email: eva.campo@uah.es, Tel.: +34 91 885 64 32, Fax: +34 91 885 5066

Class	Abbreviated name	Organism	Accessions
		**CHLOROPHYTA**	
**Prasinophyceae**	BPR	*Bathycoccus prasinos*	NC023273
	MCO	*Micromonas* sp. RCC299	NC012643
	MON	*Monomastix* sp. OKE-1	NC022797
	OTA	*Ostreococcus tauri*	NC008290
**Trebouxiophyceae**	APR	*Auxenochlorella protothecoides*	NC026009
	BBR	*Botryococcus braunii*	NC027722
	CHE	*Chlorella heliozoae*	KY629615
	CSO	*Chlorella sorokiniana*	NC024626
	CSP	*Chlorella* sp. ArM0029B	KF554428
	CSU	*Coccomyxa* sp. C-169	NC015316
	CVA	*Chlorella variabilis*	NC025413
	HEL	*Helicosporidium* sp.	NC017841
	LIN	*Lobosphaera incisa*	NC027060
	MIC	*Micractinium conductrix*	KY629619
	PCR*	*Prasiola crispa*	KR017746-7
	PWI	*Prototheca wickerhamii*	NC001613
	PZO1	*Prototheca zopfii* SAG 2021	MF197534
	PZO2	*Prototheca zopfii* SAG 2063	MF197533
	TAG*	*Trebouxia aggregata*	EU123944-9
	TR9	*Trebouxia* sp. TR9	MH917293
	TRE	*Trebouxiophyceae* sp. MX-AZ01	NC018568
**Ulvophyceae**	GSA	*Gloeotilopsis sarcinoidea*	KX306822
	PAK	*Pseudendoclonium akinetum*	NC005926
	UPE	*Ulva pertusa*	NC035722
	UPR	*Ulva prolifera*	NC028538
**Chlorophyceae**	CMO	*Chlamydomonas moewusii*	NC001872
	CRE	*Chlamydomonas reinhardtii*	NC001638
	GPE	*Gonium pectorale*	NC020437
	PST	*Pleodorina starrii*	NC021108
	TOB	*Tetradesmus obliquus*	NC002254
		**STREPTOPHYTA**	
	CAT	*Chlorokybus atmophyticus*	NC009630
	CVU	*Chara vulgaris*	NC005255
	MVI	*Mesostigma viride*	NC_008240

Table S1. List of the chlorophytes and streptophytes whose mtDNAs were compared in this study. *, Incomplete sequences.

Organism	RPL10 motif	Annotation	Positions	Length (CDS / tRNA)	Intron length	Direction	Upstream gene	Downstream gene
				**rpl10 gene**				
BPR	-	hypothetical protein CDS	21616 - 22164	549	NA	reverse	nad10	trnH (gtg)
OTA	-	ORF153 CDS	11841 - 12293	462	NA	reverse	nad10	trnH (gtg)
MCO	-	UA	113 - 622	510	NA	forward	nad10	trnH (gtg)
MON	+	orf202b	56108 - 56716	609	NA	reverse	nad10	trnY (gta)
APR	+	hypothetical protein	21089 - 21721	633	NA	forward	rps10	rps3
PWI	+	orf174 = ymf45 CDS	44191 - 44715	525	NA	forward	rps10	rps3
HEL	-	Orf160b CDS	19006 - 19488	483	NA	forward	rps10	rps3
PZO1	+	rpl10	37446 - 37967	522	NA	forward	rps10	rps3
PZO2	+	rpl10	36373 - 36894	522	NA	forward	rps10	rps3
CHE	-	UA	30566 - 31162	597	NA	forward	rps19	rrns
CSO	-	orf188	5000 - 5563	564	NA	reverse	rps19	rrns
CSP	+	orf180	44706 - 45248	543	NA	reverse	rps19	rrns
MIC	+	UA	59296 - 59844	549	NA	forward	rps19	rrns
CVA	-	UA	2404 - 3045	642	NA	reverse	rps19	rrns
BBR	-	orf203	3996 - 4607	612	2598	forward	rps19	trnR (cgt) / rrnl
CSU	+	UA	63474 - 64298	825	NA	forward	rps19	trnR (cgt) / rrnl
TRE	-	orf012	73225 - 74091	867	NA	forward	rps19	rrnl
TAG*	+	rpl10	15155 - 15685	531	NA	reverse	rps19	rpl5
TR9	+	rpl10	7587 - 8147	561	NA	forward	rps19	rpl5
LIN	+	orf27	60413 - 61084	672	NA	forward	rps19	trnE (ttc)
RCR*	-	UA	300 - 1136	837	NA	forward	?	nad3
				**tRNA genes**				
BBR	NA	trnF (gaa) / UA	30597 - 32959	72	2291	forward	trnQ (ttg)	trnK (ttt)
	NA	trnH (gug) / UA	40824 - 44243	74	3346	forward	trnS (tga)	trnG (tcc)
	NA	trnW (cca) / UA	56454 - 58011	73	1485	forward	trnC (gca)	trnE (ttc)
TRE	NA	trnG (ucc) / UA	67060 - 68774	71	1644	forward	trnH (gug)	trnP (ugg)
TAG*	NA	trnP (ugg) / UA	8283 - 10261	74	1905	reverse	trnG (ucc)	atp6
PCR**	NA	trnI (gau) / UA	22611 - 24434	74	1750	forward	nad6	trnP (ugg)
	NA	trnM (cat) / UA	14989 - 17084	74	2022	forward	trnR (ucu)	nad4
	NA	trnR (ucu) / UA	10742 - 14253	74	3560	forward	rpl6	trnM (cat)

Table S2. Unannotated tRNA genes and ORFs identified as the *rpl10* gene in the studied mitochondrial genomes. RPL10 protein motifs were searched with MotifSearch, which is available at https://www.genome.jp/tools/motif/. Algal species are arranged according to their appearance in the phylogram of Figure 2. *GenBank accessions for TAG and PCR (contig 1) are EU123949 and KR017746, respectively. ** GenBank accessions for PCR (contig 2) is KR017747. +: presence of RPL10 motif, and -: absence of RPL10 motif. NA: not applicable, UA: unannotated. ?: no available sequence upstream of the gene.

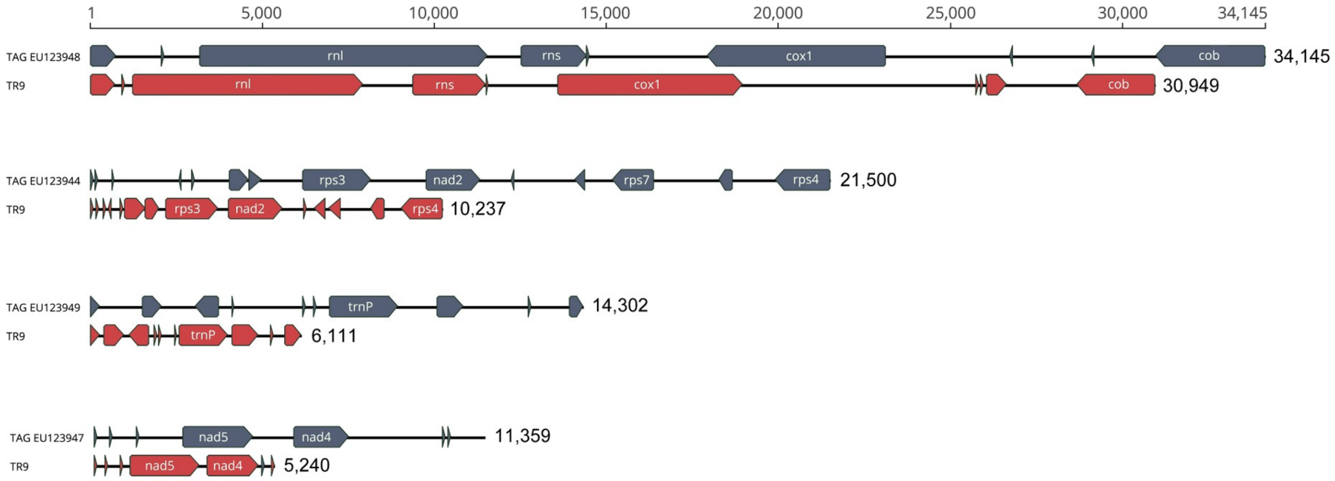

Figure S1. Genetic maps of four regions of the mtDNA of *Trebouxia aggregata* (accessions EU123944, EU123947, EU123948 and EU123949) and their counterparts in *Trebouxia* sp. TR9. Coding regions are indicated as blue and garnet arrows for *T. aggregata* (TAG) and *Trebouxia* sp. TR9 (TR9), respectively. Arrowheads indicate transcription directions. Non-coding regions are indicated by a black line.

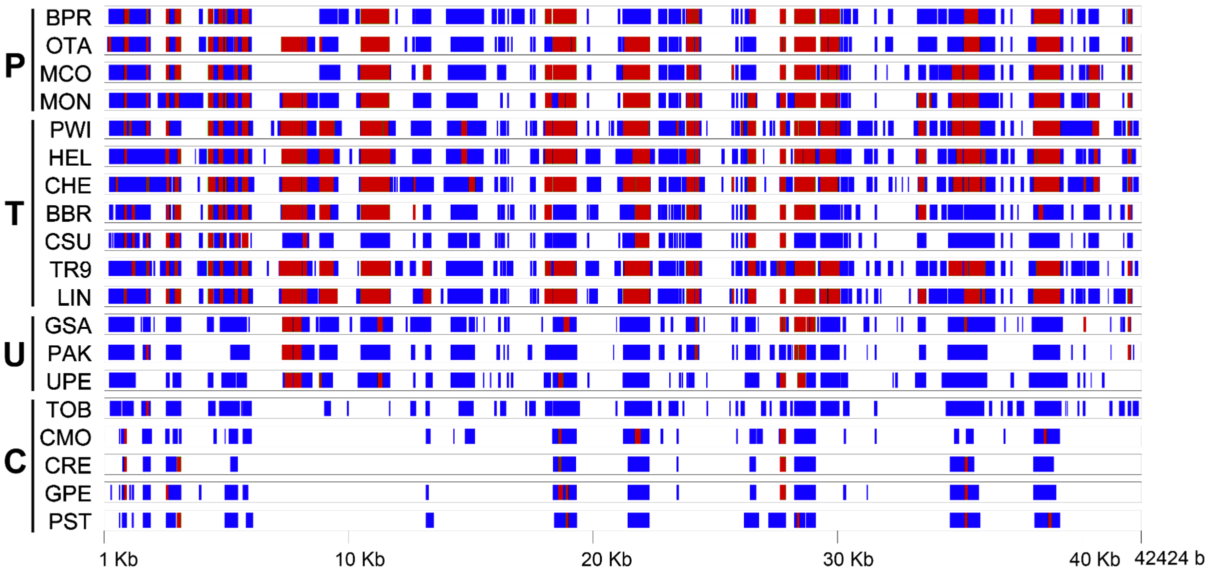

Figure S2. Whole-genome alignment of the *Trebouxia* sp. TR9 mitogenome with other chlorophyte mitochondrial genomes obtained with MultiPipMaker [68], using *Mesostigma viride* as the reference. Sequence identity is shown by red (75–100%), blue (50–75%), and white (<50%). The systematic classification is indicated on the left (P: Prasinophyceae, T: Trebouxiophyceae, U: Ulvophyceae, C: Chlorophyceae).
